# Food Preferences and Food Choice Determinants in a Polish Adolescents’ COVID-19 Experience (PLACE-19) Study

**DOI:** 10.3390/nu13082491

**Published:** 2021-07-21

**Authors:** Dominika Głąbska, Dominika Skolmowska, Dominika Guzek

**Affiliations:** 1 Department of Dietetics, Institute of Human Nutrition Sciences, Warsaw University of Life Sciences (SGGW-WULS), 02-776 Warsaw, Poland; dominika_skolmowska@sggw.edu.pl; 2 Department of Food Market and Consumer Research, Institute of Human Nutrition Sciences, Warsaw University of Life Sciences (SGGW-WULS), 02-776 Warsaw, Poland; dominika_guzek@sggw.edu.pl

**Keywords:** food preferences, food choices, Food Preference Questionnaire (FPQ), Food Choice Questionnaire (FCQ), adolescents, national study, population-based study, PLACE-19 Study

## Abstract

Food preferences are within the most important determinants of food choices; however, little is known about their complex associations, and no studies were conducted in the period of the COVID-19 pandemic. The aim of the study was to analyze the association between food preferences and food choice determinants in adolescents aged 15–20 years within the Polish Adolescents’ COVID-19 Experience (PLACE-19) Study. The PLACE-19 Study included a random quota sampling conducted in the whole of Poland and covered a population-based sample of 2448 secondary school students. The food preferences were assessed using a validated Food Preference Questionnaire (FPQ), and the food choices were assessed using a validated Food Choice Questionnaire (FCQ). The statistical analysis comprised k-means clustering and linear regression adjusted for sex and age. Four homogenous clusters of respondents were defined based on the food choice motives—“healthy eaters” (health as the most important determinant of food choices), “hedonists” (convenience, sensory appeal, and price as the most important determinants), “indifferent consumers” (low significance for all determinants), and “demanding consumers” (high significance for all determinants). The preferences for all food categories differed when comparing between clusters presenting various food choice determinants (*p* < 0.001). The “healthy eaters” were characterized by the highest preference for vegetables; the “hedonists” preferred meat/fish, dairy, and snacks; the “demanding consumers” had a high preference for all food categories, while “indifferent consumers” had a low preference for all food categories. All preference scores were positively associated with mood, convenience, sensory appeal, natural content, and price (*p* < 0.05). The results confirmed the association between food preferences and food choice determinants in adolescents, as well as allowed adolescents to be clustered into segments to define various needs and motives among the identified segments. For public health purposes, it may be crucial to educate “hedonists,” with a high preference for meat/fish, dairy and snacks, accompanied by convenience, sensory appeal, and price as the most important determinants of their food choices.

## 1. Introduction

Food choice is a broad term, which includes frequent, multifaceted, situational, dynamic, and complex decisions, which lead to food behaviors where people acquire, prepare, serve, give away, store, eat, and clean up [1]. There are multiple factors creating food choices, which are leveled into the categories of food-related features (characteristics of the product), individual differences (associated with the consumer), and society-related features (culture, economy, and related norms) [2]. Within individual differences, Chen and Antonelli [2] describe also various types of determinants, as they include personal-state factors (biological features, physiological needs, psychological components, habits, and experiences), and cognitive factors (knowledge and skills, attitude, liking and preferences, anticipated consequences, and personal identity).

In spite of multiple factors influencing food choices, the preferences are by many researchers believed to be of the highest significance and to be the most important predictor of food choices, if the availability and economic factors are not interfering [3]. Food preferences are also created by multiple factors, while both genetic and environmental ones play a role [4]. Moreover, the preferences are not stable, as prepuberty children often reject many food products which they previously enjoyed, but after puberty, they commonly begin to prefer some food products which they did not enjoy before [5]. Taking this into account, adolescence is important for food choices that are developed before that time and for food preferences that are established during this period, as their determinants are quite similar during adolescence and throughout adulthood [6].

Food preferences are determinants of food choices [7] and are also significantly associated with the followed diet [8] and its health-related consequences, including body mass [9], obesity risk [10], and gastrointestinal symptoms [11]. As a result, understanding food preferences is necessary for public health purposes, to enable adequate nutritional education to improve the general quality of diet and influence food choices [12].

When food choices are analyzed, the essential aspect is associated with the motives underlying the selection of food, including health, mood, convenience, sensory appeal, natural content, price, weight control, familiarity, and ethical concern [13]. The study conducted by Metelev [14], in a group of Russian adults, indicated that for consumers segmented based on their food preferences, their food choice motives defined the clusters in which they were grouped. However, for adolescents, such research was not conducted so far.

Moreover, there were no such studies conducted during the global COVID-19 pandemic. It may be assumed that in this period the association between food preferences and food choice determinants may have been altered due to general changes of lifestyle that are observed [15]. Additionally, specific changes in eating habits [16] and food priorities were noted during the COVID-19 pandemic [17], and they may have been associated with food choice determinants. The other issue that should be emphasized results from the still observable COVID-19 crisis, with 1.58–8.76 million related deaths predicted until the end of 2024 [18], which necessitates more studies in this specific period to recognize properly the conditions of the so-called COVID-19 era [19].

Considering this fact, the aim of the study was to analyze the association between food preferences and food choice determinants in adolescents aged 15–20 years within the Polish Adolescents’ COVID-19 Experience (PLACE-19) Study. It was hypothesized that during the COVID-19 pandemic, there is an association between food preferences and food choice determinants in adolescents and that it may allow specific educational needs of subgroups of adolescents to be identified.

## 2. Materials and Methods

### 2.1. Ethical Statement

The PLACE-19 Study was approved by the Ethics Committee of the Institute of Human Nutrition Sciences of the Warsaw University of Life Sciences, to be conducted in Polish secondary schools. All the participants and their parents/legal guardians provided their informed consents to participate and all the procedures were based on the Declaration of Helsinki.

The PLACE-19 Study was supervised by the Institute of Human Nutrition Sciences, Warsaw University of Life Sciences (WULS-SGGW), which was responsible for the data gathering and analysis within the whole project. The PLACE-19 Study was developed to assess the general situation of Polish adolescents during the COVID-19 pandemic, which included their personal protective behaviors [20,21,22] and dietary habits, as well as their motives and implications [23,24,25,26].

### 2.2. PLACE-19 Population

The PLACE-19 population included two cohorts, sampled separately to assess personal protective behaviors [20,21,22] and dietary habits [23,24,25,26] in order to reduce the burden associated with the participation in the study. Both cohorts were sampled according to the same methodology to gather representative samples of respondents. 

The cohort gathered to assess dietary habits was recruited using a random quota stratified sampling method in all regions of Poland to sample a cohort representative for regions, including all the voivodeships (administrative units in Poland). The procedure was based on the sampling of random counties within voivodeships, accompanied by the sampling of random schools within counties. The whole procedure was described in previous studies [23,24,25,26], and it was dated from 29 April 2020 to 23 May 2020. 

The sampling included inviting secondary schools to participate in the study (the local Boards of Education arranged if needed) and if the principal of the school agreed, inviting students to participate. The participation was voluntary, and it was conditioned by providing informed consent by students and by their parents/legal guardians.

The inclusion criteria were the following:

- Students of the secondary schools that were randomly selected within two stages of stratified sampling procedure (sampling counties within voivodeships and schools within counties) [23,24,25,26];
- Aged 15–20 (age attributed to a secondary level of education in Poland, while assuming the possibility of having an adolescent 1 year younger or older than a standard age of 16–19);
- Informed consent of students and of parents/legal guardians to participate.

The exclusion criteria were the following:

- Missing/unreliable data in the provided questionnaires;
- Being included to a cohort sampled to assess personal protective behaviors within the PLACE-19 Study [18,19,20].

The cohort that was sampled to assess dietary habits and was included based on the presented criteria within the PLACE-19 Study accounted for 2448 secondary school students, as described previously [23,24,25,26].

### 2.3. Questionnaires

While the PLACE-19 Study was conducted, secondary education in all schools in Poland was suspended, and a system of remote education was implemented, as decided by the Polish Ministry of Education [27]. Taking this into account, the PLACE-19 Study was conducted using a computer-assisted web interview (CAWI). A link to an electronic questionnaire was provided for all the students who were included in the study cohort, while the tool was developed to not gather any sensitive or personal data, which would allow the identification of study participants. 

The presented analysis included assessment of the food preferences, conducted while using Food Preference Questionnaire (FPQ) developed by Smith et al. [6], and assessment of the food choices, conducted while using Food Choice Questionnaire (FCQ) developed by Steptoe et al. [13].

The FPQ was developed and validated by Smith et al. [6] to obtain information about preferences of specific food products and food categories from adolescents and adults. This tool was elaborated based on the other tool, which was dedicated to assessing preferences of children only, based on the information provided by their parents [28]. The FPQ allows the assessment of preferences for the following categories of food products: vegetables, fruit, meat/fish, dairy, snacks, and starches, based on the information provided for specific food items within the listed categories (vegetables—18 items, fruit—7 items, meat/fish—12 items, dairy—10 items, snacks—9 items, starches—6 items). A total number of 62 food items are included, while each of the respondents is asked about how much on average they enjoy it. For each food item, the respondent chooses one of the following answers: (1) dislike a lot, (2) dislike a little, (3) neither like nor dislike, (4) like a little, (5) like a lot, and (6) not applicable (the last answer should be indicated for each food item that the respondent does not know, or does not remember ever having tried, while for any other they should choose the answer defining specific preference). Based on the answers provided for specific items (close-ended questions), the preferences for food categories are calculated by summing the single item preference scores within each food category and dividing this sum by the number of items [29].

The FCQ was developed and validated by Steptoe et al. [13] to obtain information about the motives underlying the selection of specific food products from adolescents and adults. The FCQ allows the assessment of the choice determinants in the following categories: health, mood, convenience, sensory appeal, natural content, price, weight control, familiarity, and ethical concern based on the answers about how important respondents consider 36 items while choosing the food they eat during a typical day. The included items describe specific features of food attributed to the listed food choice determinants (health—6 items, mood—6 items, convenience—5 items, sensory appeal—4 items, natural content—3 items, price—3 items, weight control—3 items, familiarity—3 items, and ethical concern—3 items), while for each of them, the respondent is asked about how important it is for them to consume, on a typical day, a food item that presents specific features. For each item, the respondent chooses one of the following answers: (1) not at all important, (2) a little important, (3) moderately important, and (4) very important. Each answer is attributed to a score from 1 (not at all important) to 4 (very important). Based on the answers provided for specific items (close-ended questions), food choice determinants are calculated by summing the single item scores within each determinant and dividing this sum by the number of items [13].

### 2.4. Statistical Analysis

The sample size was estimated based on the calculation for the population of Polish adolescents aged 15–20 years (2,170,464, as reported by the Central Statistical Office (CSO) in Poland [30]), at a 95% confidence level and 5% margin of error while assuming a percentage of 50%. Taking into account the presented conditions, the required sample size was calculated as 384 respondents; thus, the recruited sample of 2448 adolescents was sufficient.

The distribution was verified for its normality using the Shapiro–Wilk test, as well as based on the assessment of the skewness and kurtosis. As the distribution was not parametric for subgroups, the groups obtained based on the k-means clustering were compared using Kruskal–Wallis test by ranks, accompanied by the post hoc Tukey test. The clustering was conducted with Euclidean distance to define homogenous groups of respondents on the basis of their food choice determinants, using a method of k-means and defining the optimum number of clusters using the Elbow method. The associations between food preferences and food choice determinants in the model adjusted for sex and age were analyzed using the linear regression with the standardized β-coefficients, as the parametric distribution was observed for the total sample. 

The statistical analysis was conducted using Statistica version 13.3 (StatSoft Inc., Tulsa, OK, USA) and JASP version 0.14.0.0 (JASP Team, 2020), while the statistical significance was attributed to *p* ≤ 0.05. 

## 3. Results

The study was conducted in a group of Polish secondary school students gathered within the second phase of the PLACE-19 Study. Due to the fact that gender (males—36.6%, females—63.4%) and age differed within the studied group (age: 15—12.1%, 16—30.8%, 17—32.6%, 18—18.2%, 19—5.7%, 20—0.5%), these indicated factors were taken into account and included to the model within the further analysis.

Food choice determinants for subgroups were stratified based on the clustering of the assessed FCQ scores within the population of the second phase of the PLACE-19 Study, and they are presented in Table 1. When the k-means algorithm was used, based on food choice determinants, four homogenous clusters of respondents were defined, and based on their general characteristics, they were described as follows: “healthy eaters” (cluster 1—health as the most important determinant of food choices); “hedonists” (cluster 2—convenience, sensory appeal, and price as the most important determinants); “indifferent consumers” (cluster 3—low significance for all determinants); “demanding consumers” (cluster 4—high significance for all determinants). While comparing determinants, the differences between clusters were taken into account, while for the majority of determinants, the high values were defined as scored ≥ 3 (described by respondents as moderately important to very important).

Food preferences were assessed by using FPQ for subgroups that were stratified based on the clustering of the assessed FCQ scores within the population of the second phase of the PLACE-19 Study; they are presented in Table 2. The preferences for all food categories differed when comparing between clusters presenting various food choice determinants. The “healthy eaters” (cluster 1) were characterized by the highest preference for vegetables; the “hedonists” (cluster 2) preferred meat/fish, dairy, and snacks; the “demanding consumers” (cluster 4) preferred all food categories, while “indifferent consumers” (cluster 3) were characterized by the low preference for all food categories.

Analysis of association between food choice determinants assessed by FCQ and vegetable preference assessed by FPQ, in a model adjusted for sex and age within the population of the second phase of the PLACE-19 Study, is presented in Table 3. The vegetable preference score was positively associated with all food choice determinants, except for familiarity. The health and natural content explained the largest amount of variance (R^2^ = 0.039, *p* < 0.001 and R^2^ = 0.034, *p* < 0.001, respectively) for the studied population.

Analysis of association between food choice determinants assessed by FCQ and fruit preference assessed by FPQ, in a model adjusted for sex and age within the population of the second phase of the PLACE-19 Study, is presented in Table 4. Fruit preference score was positively associated with all food choice determinants. The health and sensory appeal explained the largest amount of variance (R^2^ = 0.031, *p* < 0.001 and R^2^ = 0.028, *p* < 0.001, respectively) for the studied population.

Analysis of association between food choice determinants assessed by FCQ and meat/fish preference assessed by FPQ, in a model adjusted for sex and age within the population of the second phase of the PLACE-19 Study, is presented in Table 5. Meat/fish preference score was positively associated with health, mood, convenience, sensory appeal, natural content, and price. The sensory appeal and price explained the largest amount of variance (R^2^ = 0.068, *p* < 0.001 and R^2^ = 0.067, *p* < 0.001, respectively) for the studied population.

Analysis of association between food choice determinants assessed by FCQ and dairy preference assessed by FPQ, in a model adjusted for sex and age, within the population of the second phase of the PLACE-19 Study, is presented in Table 6. Dairy preference score was positively associated with all food choice determinants, except for weight control. The sensory appeal and price explained the largest amount of variance (R^2^ = 0.039, *p* < 0.001 and R^2^ = 0.035, *p* < 0.001, respectively) for the studied population.

Analysis of association between food choice determinants assessed by FCQ and snacks preference assessed by FPQ, in a model adjusted for sex and age within the population of the second phase of the PLACE-19 Study, is presented in Table 7. Snack preference score was positively associated with all food choice determinants, except for health and ethical concerns. The convenience and sensory appeal explained the largest amount of variance (R^2^ = 0.21 *p* < 0.001 for both determinants) for the studied population.

Analysis of association between food choice determinants assessed by FCQ and starches preference assessed by FPQ, in a model adjusted for sex and age within the population of the second phase of the PLACE-19 Study, is presented in Table 8. Starches preference score was positively associated with all food choice determinants. The health, sensory appeal and mood explained the largest amount of variance (R^2^ = 0.033, *p* < 0.001; R^2^ = 0.028, *p* < 0.001 and R^2^ = 0.027, *p* < 0.001, respectively) for the studied population.

## 4. Discussion

The conducted analysis allowed identifying specific clusters of adolescent food consumers according to their food preferences and food choice determinants to define various needs and motives among the identified segments. Indicating such segments of consumers may be crucial for their nutritional education and to obtain an effective change of their dietary patterns into health-promoting ones, as the nutritional behaviors are determined by the preferences and choices [31]. 

The clusters defined within the conducted study for Polish adolescents were similar to those formulated by Metelev in a group of Russian adults [14], in which a similar approach was applied to cluster respondents. It may be indicated that in the presented study, two clusters were characterized by their preferences similar for all the products and choice determinants similar for all determinants, but of the opposite direction—either a high preference of all food categories and the important role of all determinants (“demanding consumers”) or a low preference of all food categories and no important role of all determinants (“indifferent consumers”). At the same time, two clusters had their preferences and choice determinants differentiated—either a high preferences of vegetables, but not the other food groups, accompanied by health as the most important determinant of food choices with lower importance of the other determinants (“healthy eaters”) or a high preferences of meat/fish, dairy, and snacks, but not the other food groups, accompanied by convenience, sensory appeal, and price as the most important determinant of food choices with lower importance of the other determinants (“hedonists”). Taking this into account, the indicated clusters may be interpreted two by two—separately as “demanding consumers” and “indifferent consumers,” and separately as “healthy eaters” and “hedonists,” corresponding to similar clusters defined by Metelev [14] defined as “restricted consumers,” “food indifferent,” “healthy eaters,” and “hedonists.”

The “demanding consumers,” as a cluster of high expectations and taking into account multiple attributes of quality, are also indicated in other studies. This cluster was similarly defined by Sajdakowska and Tekień [32] in the study of Polish consumers of yogurt as “quality-oriented”/”quality-enthusiast” ones, indicating many of their food choice determinants, including health-promoting value, sensory features, availability on the market, and production by traditional methods, as important determinants of choice. However, as indicated by other authors, this approach is not representative of the general population and is typical for respondents with a higher level of knowledge and motivation [33]. It may be supposed that nutritional knowledge, followed by motivation to follow a properly balanced and sustainable diet, may set the conditions for this cluster of consumers, as indicated by other authors that having higher concerns about food sustainability dimensions, such as ethical and environmental issues and local production, appear to be associated with having a healthier diet [34].

On the other hand, “indifferent consumers”, as a cluster characterized by declared low importance of all determinants of choice, may be defined as those with a low interest in food products and their specific attributes, thus; they are characterized by a low willingness to pay for those attributes [35]. As a result, such consumers may not read food labels [36], may be not interested in food safety issues [37], and may not be oriented toward any specific aspect of food products, including their health-promoting properties [38]. It may be assumed that they do not have any specific expectations associated with food products, as the major value of food for them is associated with satisfying their hunger, rather than palatability and pleasure of consumption, but this approach is rare [39]. It may result from the fact that they have neither positive nor negative expectations associated with food products, but they have neutral expectations associated with choosing minimally tolerable products as good enough for them [40].

The clusters described above presented a unified approach toward all food choice determinants and a unified approach toward all food products groups, as they were for them either of high importance (“demanding consumers”) or of low importance (“indifferent consumers”). Simultaneously, “healthy eaters” and “hedonists” present their approach diversified, depending on the product group and attribute. 

The “healthy eaters” are a cluster formulated also by other authors as “health-driven consumers”, who try to follow a healthy and balanced diet and choose products of health-promoting values [41]. Such consumers may be characterized by increased interest in the energy value of food products [42], and this approach may be associated with the choice of specific food products, as the health-oriented consumers may consciously choose products presenting higher health-promoting value [43]. In the presented study, it was observed that they more often choose healthy food products, and they also indicated them as preferred ones, as it was stated for vegetables. 

The “hedonists” are indicated by other authors as those who are motivated while choosing food products mainly by their mood and price of food products [44], which is in agreement with the results of this study with convenience, sensory appeal, and price of food products as the most important determinants. They may be more likely to use convenience outlets that may support unhealthy food promotion [45]. When comparing them with other consumers, this cluster may present general poor nutritional diet quality, as it is not within their priorities [46]. Such an approach corresponds to this study with a higher preference for meat/fish, dairy, and snacks.

In the studied group of adolescents in the linear regression analysis, all preference scores were positively associated with mood, convenience, sensory appeal, natural content, and price. While analyzing indicated determinants, it should be emphasized that they were especially important neither for “indifferent consumers” nor for “healthy eaters” but only for “demanding consumers” and “hedonists.” However, in the case of “demanding consumers,” all attributes were indicated as important, while for “hedonists,” specifically convenience, sensory appeal, and price were stated to be most important. Taking this into account, the obtained results confirmed the association between food preferences and food choice determinants in adolescents and allowed defining clusters that are crucial for public health purposes. The “hedonists” with a high preference for meat/fish, dairy, and snacks, accompanied by convenience, sensory appeal, and price of food products as the most important determinants of their food choices, may be indicated as a cluster that should be educated in particular. For this cluster, modifying food choice determinants, which may be obtained by increased nutritional knowledge, may influence also preferences and the choices of food products, as it favors the adoption of dietary recommendations [47]. In spite of the fact that such actions are difficult, they are needed to improve the quality of the diet of adolescents, as indicated by the World Health Organization (WHO) within the most important issues and challenges for the health sector [48].

In spite of the fact that the conducted study provided novel observations gathered during the COVID-19 pandemic, some limitations of the study must be listed. The most important one is associated with the general recall bias, resulting from the fact that respondents may have reported inaccurate answers, either due to their inability to recognize their preferences and food choice determinants or due to their willingness to be perceived in the other way than they are. The other limitation is associated with the specific population in which the study was conducted, being only the population of adolescents during the COVID-19 pandemic and including only one country. Last but not least, it should be indicated that some potential influencing factors were not taken into account, such as the educational level of parents or family income, which may have also influenced food choices.

## 5. Conclusions

The obtained results confirmed the association between food preferences and food choice determinants in adolescents, as well as allowed clustering adolescents according to their food preferences and food choice determinants to define various needs and motives among the identified segments. It was stated that for public health purposes, it may be crucial to educate a cluster named “hedonists” (with a high preference of meat/fish, dairy, and snacks, accompanied by convenience, sensory appeal, and price as the most important determinants of their food choices).

## Figures and Tables

**Table 1:** The food choice determinants for subgroups were stratified based on the clustering of the scores assessed while using the Food Choice Questionnaire (FCQ) within the population of the second phase of the Polish Adolescents’ COVID-19 Experience (PLACE-19) Study (*n* = 2448).

Food Choice Determinants	Cluster 1 (Healthy Eaters) (*n* = 763)		Cluster 2 (Hedonists) (*n* = 455)		Cluster 3 (Indifferent Consumers) (*n* = 590)		Cluster 4 (Demanding Consumers) (*n* = 640)		*p*
	Mean ± SD	Median (Min–Max)	Mean ± SD	Median (Min–Max)	Mean ± SD	Median (Min–Max)	Mean ± SD	Median (Min–Max)	
Health	3.1 ± 0.5	3.0 * (2.7–3.5) ^a^	2.2 ± 0.6	2.2 * (1.8–2.7) ^a b^	1.8 ± 0.5	2.0 * (1.3–2.0) ^c^	3.6 ± 0.4	3.7 * (3.2–4.0) ^b^	<0.001
Mood	2.6 ± 0.6	2.5 * (2.2–3.0) ^a^	2.5 ± 0.7	2.5 * (2.0–3.0) ^b^	1.7 ± 0.5	1.8 * (1.3–2.0) ^c^	3.5 ± 0.5	3.5 * (3.0–4.0) ^d^	<0.001
Convenience	2.5 ± 0.6	2.6 * (2.2–3.0) ^a^	3.2 ± 0.6	3.2 * (2.8–3.8) ^a^	1.9 ± 0.5	2.0 * (1.4–2.0) ^b^	3.5 ± 0.5	3.6 * (3.0–4.0) ^c^	<0.001
Sensory appeal	2.9 ± 0.6	3.0 * (2.5–3.3) ^a^	3.2 ± 0.6	3.3 * (2.8–3.5) ^b^	2.0 ± 0.6	2.0 * (1.8–2.3) ^b^	3.6 ± 0.4	3.8 * (3.3–4.0) ^d^	<0.001
Natural content	3.0 ± 0.6	3.0 * (2.7–3.3) ^a^	1.9 ± 0.6	2.0 * (1.3–2.3) ^b^	1.7 ± 0.5	1.7 * (1.0–2.0) ^c^	3.5 ± 0.5	3.7 * (3.0–4.0) ^d^	<0.001
Price	2.7 ± 0.7	2.7 * (2.3–3.0) ^a^	3.2 ± 0.7	3.3 * (3.0–4.0) ^b^	1.9 ± 0.6	2.0 * (1.7–2.0) ^c^	3.5 ± 0.5	3.7 * (3.0–4.0) ^d^	<0.001
Weight control	2.9 ± 0.7	3.0 * (2.3–3.3) ^a^	1.8 ± 0.7	1.7 * (1.0–2.3) ^b^	1.7 ± 0.6	1.7 * (1.0–2.0) ^c^	3.4 ± 0.6	3.3 * (3.0–4.0) ^d^	<0.001
Familiarity	2.2 ± 0.6	2.3 * (1.7–2.7) ^a^	2.4 ± 0.7	2.3 * (2.0–3.0) ^b^	1.7 ± 0.5	1.7 * (1.3–2.0) ^b^	3.3 ± 0.6	3.3 * (3.0–4.0) ^c^	<0.001
Ethical concern	2.0 ± 0.7	2.0 * (1.7–2.7) ^a^	1.5 ± 0.5	1.3 * (1.0–2.0) ^b^	1.5 ± 0.5	1.3 * (1.0–2.0) ^c^	3.1 ± 0.8	3.0 * (2.7–4.0) ^d^	<0.001

* not parametric distribution (*p* ≤ 0.05 for Shapiro–Wilk test); ^a, b, c, d^—values marked with different letters in rows differ significantly.

**Table 2:** The food preferences were assessed by using the Food Preference Questionnaire (FPQ) for subgroups stratified based on the clustering of the scores assessed while using the Food Choice Questionnaire (FCQ) within the population of the second phase of the Polish Adolescents’ COVID-19 Experience (PLACE-19) Study (*n* = 2448).

Food Category	Cluster 1 (Healthy Eaters) (*n* = 763)		Cluster 2 (Hedonists) (*n* = 455)		Cluster 3 (Indifferent Consumers) (*n* = 590)		Cluster 4 (Demanding Consumers) (*n* = 640)		*p*
	Mean ± SD	Median (Min–Max)	Mean ± SD	Median (Min–Max)	Mean ± SD	Median (Min–Max)	Mean ± SD	Median (Min–Max)	
Vegetable	3.8 ± 0.7	3.9 * (3.4–4.3) ^a^	3.7 ± 0.7	3.8 * (3.2–4.2) ^b^	3.5 ± 0.8	3.6 * (2.9–4.1) ^c^	3.8 ± 0.8	4.0 * (3.4–4.4) ^a^	<0.001
Fruit	4.4 ± 0.7	4.6 * (4.0–5.0) ^a^	4.4 ± 0.7	4.6 * (4.0–4.9) ^a^	4.0 ± 1.0	4.1 * (3.6–5.0) ^b^	4.4 ± 0.8	4.7 * (4.1–5.0) ^c^	<0.001
Meat/fish	3.4 ± 0.8	3.5 * (2.9–4.0) ^a b^	3.5 ± 0.8	3.6 * (3.1–4.0) ^a^	3.3 ± 0.9	3.4 * (2.8–4.0.) ^b^	3.5 ± 0.8	3.6 * (2.9–4.1) ^a^	<0.001
Diary	3.6 ± 0.7	3.7 * (3.2–4.1) ^a^	3.8 ± 0.7	3.9 * (3.4–4.3) ^b^	3.5 ± 0.8	3.6 * (3.0–4.1) ^a^	3.8 ± 0.8	3.9 * (3.3–4.3) ^b^	<0.001
Snacks	4.1 ± 0.8	4.1 * (3.7–4.7) ^a^	4.4 ± 0.6	4.6 * (4.1–4.9) ^b^	4.0 ± 0.9	4.1 * (3.6–4.8) ^a^	4.2 ± 0.8	4.3 * (3.9–4.9) ^c^	<0.001
Starches	4.1 ± 0.7	4.2 * (3.8–4.7) ^a b^	4.1 ± 0.7	4.2 * (3.7–4.6) ^b^	3.8 ± 0.9	4.0 * (3.2–4.5) ^c^	4.2 ± 0.8	4.3 * (3.8–4.8) ^a^	<0.001

* not parametric distribution (*p* ≤ 0.05 for Shapiro–Wilk test); ^a, b, c^—values marked with different letters in rows differ significantly.

**Table 3:** Analysis of association between food choice determinants assessed by Food Choice Questionnaire (FCQ) and vegetable preference assessed by Food Preference Questionnaire (FPQ) (model adjusted for sex and age) within the population of the second phase of the Polish Adolescents’ COVID-19 Experience (PLACE-19) Study (*n* = 2415).

Food Choice Determinants	Unstandardized Coefficients		Standardized Coefficients β	*p*	R^2^
	β	Standard Error			
Health	0.153	0.016	0.191	<0.001	0.039
Mood	0.111	0.017	0.134	<0.001	0.021
Convenience	0.088	0.017	0.106	<0.001	0.015
Sensory appeal	0.130	0.018	0.146	<0.001	0.025
Natural content	0.136	0.016	0.179	<0.001	0.034
Price	0.115	0.016	0.141	<0.001	0.023
Weight control	0.075	0.015	0.102	<0.001	0.014
Familiarity	0.031	0.017	0.037	0.067	0.005
Ethical concern	0.078	0.016	0.100	<0.001	0.014

**Table 4:** Analysis of association between food choice determinants assessed by Food Choice Questionnaire (FCQ) and fruit preference assessed by Food Preference Questionnaire (FPQ) (model adjusted for sex and age) within the population of the second phase of the Polish Adolescents’ COVID-19 Experience (PLACE-19) Study (*n* = 2330).

Food Choice Determinants	Unstandardized Coefficients		Standardized Coefficients β	*p*	R^2^
	β	Standard Error			
Health	0.116	0.015	0.162	<0.001	0.031
Mood	0.100	0.015	0.136	<0.001	0.024
Convenience	0.073	0.015	0.098	<0.001	0.016
Sensory appeal	0.121	0.017	0.151	<0.001	0.028
Natural content	0.084	0.014	0.124	<0.001	0.021
Price	0.067	0.015	0.092	<0.001	0.015
Weight control	0.053	0.014	0.082	<0.001	0.013
Familiarity	0.046	0.015	0.062	0.003	0.010
Ethical concern	0.049	0.014	0.071	<0.001	0.011

**Table 5:** Analysis of association between food choice determinants assessed by Food Choice Questionnaire (FCQ) and meat/fish preference assessed by Food Preference Questionnaire (FPQ) (model adjusted for sex and age) within the population of the second phase of the Polish Adolescents’ COVID-19 Experience (PLACE-19) Study (*n* = 2425).

Food Choice Determinants	Unstandardized Coefficients		Standardized Coefficients β	*p*	R^2^
	β	Standard Error			
Health	0.081	0.019	0.087	<0.001	0.066
Mood	0.071	0.019	0.075	<0.001	0.064
Convenience	0.068	0.019	0.071	<0.001	0.064
Sensory appeal	0.101	0.020	0.099	<0.001	0.068
Natural content	0.064	0.018	0.073	<0.001	0.064
Price	0.087	0.018	0.093	<0.001	0.067
Weight control	0.009	0.017	0.011	0.601	0.059
Familiarity	0.011	0.019	0.012	0.554	0.059
Ethical concern	0.030	0.018	0.034	0.088	0.060

**Table 6:** Analysis of association between food choice determinants assessed by Food Choice Questionnaire (FCQ) and dairy preference assessed by Food Preference Questionnaire (FPQ) (model adjusted for sex and age) within the population of the second phase of the Polish Adolescents’ COVID-19 Experience (PLACE-19) Study (*n* = 2417).

Food Choice Determinants	Unstandardized Coefficients		Standardized Coefficients β	*p*	R^2^
	β	Standard Error			
Health	0.064	0.017	0.079	<0.001	0.023
Mood	0.095	0.017	0.114	<0.001	0.029
Convenience	0.109	0.017	0.130	<0.001	0.033
Sensory appeal	0.136	0.018	0.151	<0.001	0.039
Natural content	0.036	0.016	0.046	0.026	0.019
Price	0.112	0.017	0.136	<0.001	0.035
Weight control	−0.010	0.015	−0.014	0.498	0.017
Familiarity	0.099	0.017	0.116	<0.001	0.030
Ethical concern	0.063	0.016	0.080	<0.001	0.023

**Table 7:** Analysis of association between food choice determinants assessed by Food Choice Questionnaire (FCQ) and snacks preference assessed by Food Preference Questionnaire (FPQ) (model adjusted for sex and age) within the population of the second phase of the Polish Adolescents’ COVID-19 Experience (PLACE-19) Study (*n* = 2341).

Food Choice Determinants	Unstandardized Coefficients		Standardized Coefficients β	*p*	R^2^
	β	Standard Error			
Health	−0.022	0.015	−0.031	0.148	0.004
Mood	0.074	0.016	0.099	<0.001	0.013
Convenience	0.104	0.016	0.136	<0.001	0.021
Sensory appeal	0.111	0.017	0.137	<0.001	0.021
Natural content	−0.054	0.015	−0.078	<0.001	0.009
Price	0.075	0.015	0.102	<0.001	0.013
Weight control	−0.062	0.014	-0.093	<0.001	0.011
Familiarity	0.084	0.016	0.111	<0.001	0.015
Ethical concern	−0.016	0.015	−0.023	0.264	0.004

**Table 8:** Analysis of association between food choice determinants assessed by Food Choice Questionnaire (FCQ) and starches preferences assessed by Food Preference Questionnaire (FPQ) (model adjusted for sex and age) within the population of the second phase of the Polish Adolescents’ COVID-19 Experience (PLACE-19) Study (*n* = 2368).

Food Choice Determinants	Unstandardized Coefficients		Standardized Coefficients β	*p*	R^2^
	β	Standard Error			
Health	0.143	0.016	0.184	<0.001	0.033
Mood	0.131	0.017	0.163	<0.001	0.027
Convenience	0.101	0.017	0.125	<0.001	0.016
Sensory appeal	0.143	0.018	0.166	<0.001	0.028
Natural content	0.106	0.016	0.143	<0.001	0.020
Price	0.114	0.016	0.145	<0.001	0.022
Weight control	0.086	0.015	0.122	<0.001	0.015
Familiarity	0.055	0.017	0.068	<0.001	0.005
Ethical concern	0.068	0.015	0.091	<0.001	0.009
